# The tiger who came to T2T: Telomere-to-Telomere genome assembly of the Sumatran tiger (*Panthera tigris sumatrae*) using nanopore simplex reads

**DOI:** 10.1186/s12864-026-12703-0

**Published:** 2026-03-21

**Authors:** Laura Louise Dean, Nadine Holmes, Phillipa Dobbs, Matthew Loose

**Affiliations:** 1 School of Life Sciences, The University of Nottingham, University Park, Nottingham NG7 2RD UK; 2 Twycross Zoo, Atherstone, Warwickshire CV9 3PX UK

**Keywords:** Genome assembly, ONT, Sumatran tiger, Hifiasm, Genomics

## Abstract

**Background:**

Chromosome-level de novo genome assemblies are vital for many aspects of biological research. Despite technological progress, achieving telomere-to-telomere assemblies remains challenging. Long-read data are critical for a contiguous genome assembly, since repeat regions cannot be assembled without them. Researchers typically generate substantial quantities of data from multiple sequencing platforms to achieve accurate and contiguous telomere-to-telomere (T2T) assemblies. This makes the process costly and complicated. Recent advances in algorithms designed to optimise assembly with long-read data have the potential to rectify this issue. Using only Oxford Nanopore Technology (ONT) simplex long-read data from the Sumatran tiger (*Panthera tigris sumatrae*), we evaluate leading methods for error correction of long-reads (NextDenovo, HERRO and hifiasm ONT) and various assembly approaches.

**Results:**

We show that correcting errors in ONT long-reads during assembly greatly improves the quality and contiguity of the resulting assembly, suggesting these methods will make high quality genome assemblies more achievable with less data. We also present the first, almost complete, T2T, *de novo* genome assembly for the Sumatran tiger, with a single technology.

**Conclusions:**

This assembly is a novel resource for genomic research and conservation efforts.

**Supplementary Information:**

The online version contains supplementary material available at 10.1186/s12864-026-12703-0.

## Background

Obtaining chromosome-level de novo genome assemblies greatly enhances studies of evolutionary biology and population genomics and is critical for conservation. Constructing telomere to telomere (T2T) assemblies still typically involves data from multiple sequencing platforms and thus remains costly, time consuming and complicated [1]. Long-read data are critical for T2T genome assembly because reads must be long enough to span long repetitive regions in a genome, to assemble through them. Oxford Nanopore Technology (ONT) is currently the leading sequencing technology for generating the longest continuous sequencing reads, with individual reads having the potential to exceed 4 Mb in length. It is the only technology capable of generating reads long enough to span certain centromeric repeat regions and segmental duplications [2]. However, standard simplex (R10) ONT long reads still have a relatively high error rate (est. 2% [3]) and so have historically not been accurate enough for stand-alone genome assembly. Typically, ONT reads are generated alongside Pacific Biosciences High Fidelity (PacBio HIFI) long reads (10–20 kb in length) or Illumina short reads (150–300 bp in length), both of which have an error rate close to 0.1% [4, 5]. ONT duplex reads can achieve read lengths similar to ONT simplex reads, but with an error rate which is on-par with that of Pac-Bio HIFI and Illumina sequencing, which makes them promising for the possibility of single-sequencing-technology genome assembly [6]. However, duplex sequencing is challenging to implement and requires large quantities of input DNA, so is probably not a viable mass- solution.

A recent, and promising advance in assembly methods is the inclusion of algorithms designed to accommodate the profile of long-read data [7]. One such method is HERRO, a deep-learning model that uses informative positions that vary between haplotypes or segments in duplications to improve the accuracy of simplex ONT reads by up to 100 fold [8]. HERRO is, however, computationally expensive and requires GPU processing, which makes it less universally accessible. Another method for correcting then assembling ONT reads is NextDenovo [9], which uses read overlap and the k-mer score chain (KSC) algorithm [10] to correct errors in long reads before assembling them. NextDenovo is also computationally intensive, although it does not require GPU. Another very recently developed method for error correction is Hifiasm (ONT), which utilises read phasing to identify and correct sequencing errors, and is considerably more computationally efficient than HERRO or NextDenovo [11]. These methods have the potential to reduce the need for multiple sequencing technologies, and thus the sequencing cost of generating T2T assemblies. We tested the efficacy of these tools to generate a de novo genome assembly using data from a single sequencing technology – ONT R10 long reads. We validate the resulting assemblies using high accuracy ONT duplex reads, existing Felidae genome assemblies and previously published Hi-C data. Our results show that assembly with error corrected simplex ONT reads is a substantial improvement on assembly without error correction and comparable to assembly with data from multiple sequencing technologies.

We use the ONT data to construct highly contiguous telomere to telomere chromosome-level genome assemblies of the Sumatran tiger (*Panthera tigris sumatrae*). The Sumatran tiger is endemic to the Indonesian island of Sumatra and although population estimates are ongoing, current assessments suggest fewer than 500 individuals likely remain in the wild [12]. There is some debate over whether the Sumatran tiger should be classified as a unique subspecies or whether it should fall under the broader subspecies *Panthera tigris sondaica*. Morphological evidence tends to point to the latter [13], whereas genomic evidence suggests the Sumatran tiger forms a distinct and monophyletic clade that should be recognised as a subspecies in its own right [14]. We chose to use the more specific nomenclature for future compatibility. *Panthera tigris* is classified as endangered at the species-level [15], and given the estimated size of the wild population in Sumatra, the Sumatran tiger would likely be critically endangered as a subspecies. We hope that this work will provide a crucial resource for conservation efforts.

## Results

### Chromosome-level genome assembly

We generated 158 Gb ONT simplex R10 data (~ 64X coverage), which comprised 10,102,284 raw reads with an N50 of 42 Kb from four ONT simplex flowcells. We also ran three duplex flowcells, which generated a total of 27 Gb duplex data (~ 11X coverage), comprising 2,870,273 raw duplex reads with an N50 of 19 kb. See Figure S1 for read length histograms.

**Fig. 1:**
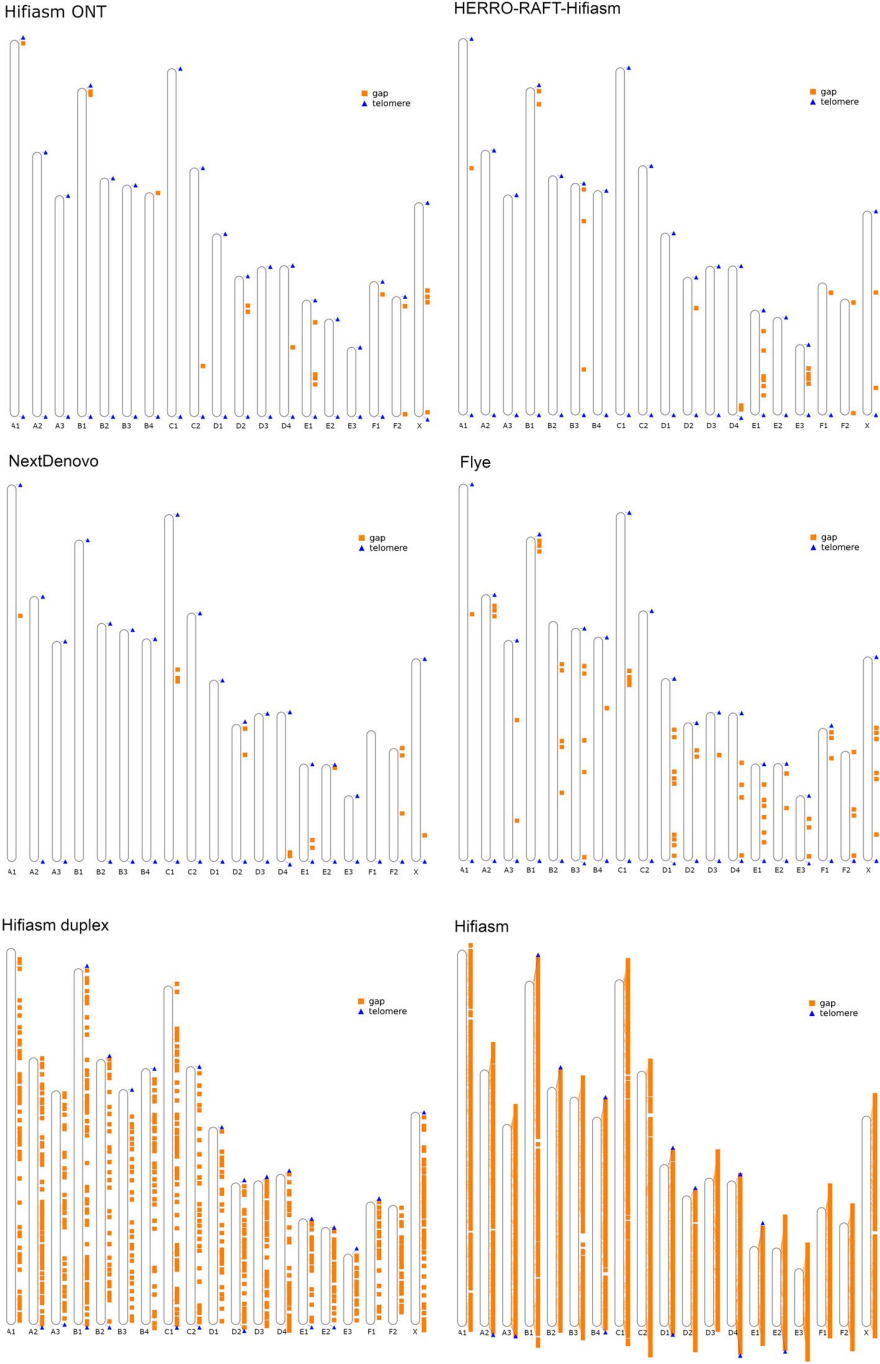
Locations of telomeric repeat sequences and gaps in the assemblies generated in this study. All assemblies were scaffolded based on homology to the published tiger assembly. Unplaced contigs were removed for clarity

We generated a benchmark tiger assembly using hifiasm with uncorrected simplex reads to compare with other assembly methods. We compared this assembly to most contiguous published domestic cat assembly (AnAms1.0 [16]) and the only other published *Panthera tigris* assembly (P.tigris_Pti1_mat1.1, a haplome assembly generated from an F1 liger [17]). The benchmark assembly was considerably more fragmented (Table 1, Fig. 1, Figure S2) and incomplete (Table 2 and Table S1) than the domestic cat and tiger haplome assemblies, as well as the other assemblies we generated.

**Table 1:** Assembly contig statistics

Assembly	Total length (bp)	Number of contigs	N50	N90	Longest contig (bp)	Shortest contig (bp)	GC content (%)	QV score	Estimated error rate
Domestic cat reference	2,462,412,258	401	21,291,731	5,730,844	75,246,234	100,606	41.87	45.8	0.00003
Tiger Haplome	2,407,136,734	115	74,391,967	28,206,420	166,184,669	101,836	41.64	47.5	0.00002
Hifiasm	1,694,903,205	4,023	655,267	170,157	6,150,673	100,064	40.97	33.0	0.00050
Hifiasm duplex	2,498,525,531	856	6,795,188	1,633,985	25,894,061	100,247	41.77	38.8	0.00013
Flye	2,403,159,788	89	64,883,702	20,032,933	197,106,460	102,535	41.66	44.3	0.00004
NextDenovo	2,412,775,875	40	139,803,469	59,657,662	204,184,323	184,266	41.66	44.0	0.00004
HERRO-RAFT-hifiasm	2,451,760,258	94	139,794,700	28,923,347	220,606,598	110,731	41.80	49.2	0.00001
Hifiasm ONT	2,462,500,948	55	142,172,726	51,966,967	238,981,921	111,431	41.91	47.9	0.00002

All assembly statistics are shown for un-scaffolded assemblies (i.e. for published assemblies, scaffolds are broken at Ns into contigs), filtered to remove fragments < 100 kb. *bp* base pairs. Published QV score and estimated error rates, also calculated using Merqury, are shown for the domestic cat reference [16] and for the tiger haplome [17] for comparison

**Table 2:** Comparison of assemblies to published reference genomes

Genome statistics relative to the domestic cat reference assembly (AnAms1.0)						
Assembly	Genome fraction (%)	NGA50	Misassemblies (#)	Mismatches per 100 kb (#)	Indels per 100 kb (#)	Liftoff genes successfully transferred
Tiger Haplome	92.80	263,698	23,267	2,145.03	331.77	20,833
Hifiasm	63.79	89,082	17,039	2,144.62	371.53	14,572
Hifiasm duplex	90.10	251,333	25,155	2,155.44	343.68	20,274
Flye	92.80	265,614	23,138	2,143.84	331.46	20,830
NextDenovo	92.83	264,291	23,749	2,145.36	331.64	20,837
HERRO-RAFT-hifiasm	92.84	263,677	25,234	2,150.70	331.57	20,854
Hifiasm ONT	92.83	264,529	25,595	2,149.62	332.07	20,850

Genome comparison to the tiger haplome assembly (P.tigris_Pti1_mat1.1)						
Assembly	Genome fraction (%)	NGA50	Misassemblies (#)	Mismatches per 100 kb (#)	Indels per 100 kb (#)	
Domestic cat reference	95.55	285,739	23,860	2,146.71	332.58	
Hifiasm	68.83	284,461	2,013	164.17	105.31	
Hifiasm duplex	96.82	2,136,330	3,405	146.97	65.74	
Flye	99.62	3,329,921	2,017	119.07	51.27	
NextDenovo	99.72	3,321,613	2398	120.5	53.52	
HERRO-RAFT-hifiasm	99.73	3,372,831	3,269	134.48	49.22	
Hifiasm ONT	99.71	3,365,310	3,335	135.54	51.83	

Genome fraction: The percentage of total bases in the reference genome that are aligned to the compared assembly. NGA50: length of aligned blocks such that longer or equal length blocks covers 50% of the reference genome. #: number

**Fig. 2:**
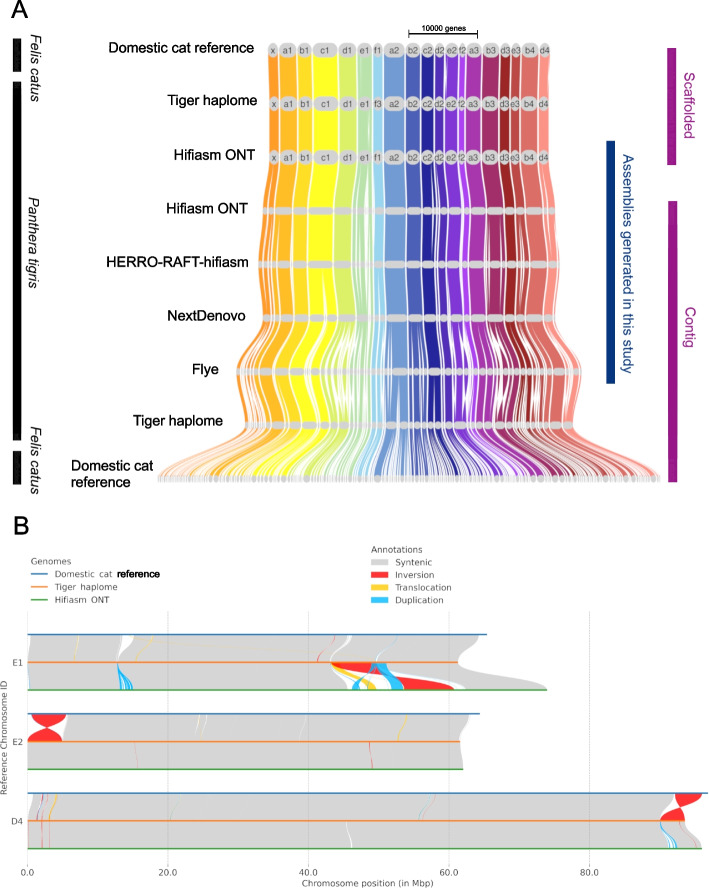
**A** Synteny plot [49] showing collinearity of published, scaffolded domestic cat (*Felis catus*) and tiger (*Panthera tigris*) haplome assemblies, alongside the hifiasm ONT assembly generated in this study, scaffolded based on homology to the tiger haplome assembly, and contig-level assemblies generated in this study (contig assemblies generated with hifiasm without the ONT mode are omitted for clarity but shown in Figure S5). The plot also shows contig-level published domestic cat and tiger haplome genomes (broken into contigs), for comparison. **B** Chromosomal rearrangements between domestic cat, tiger haplome and hifiasm ONT assemblies on selected chromosomes

We tested assembly including the duplex reads alongside simplex reads. This was an improvement on the benchmark hifiasm assembly, but it remained more fragmented and incomplete than the other assemblies (Table 1, Table 2, Table S1, Fig. 1, Fig. 2). Next, we tested assembly with Flye, which is designed for long error-prone reads. Flye generated a reasonably good assembly with only the uncorrected simplex reads. The Flye assembly was very similar to the published tiger haplome assembly in most of the measures we used to assess genome quality, and was let down largely by shorter contig lengths (Table 1) and consequently, a lack of T2T chromosomes at the contig-level (Table 3). Finally, we tested three assembly methods that involved pre-assembly error correction of the simplex reads: one using NextDenovo, one following a RAFT-HERRO-hifiasm pipeline (see methods for more details) and one using Hifiasm ONT. Primary assemblies were used for comparisons and details on partially phased haplotype assemblies are given in Table S2. The assemblies generated using error corrected simplex reads were by far the most contiguous, with Hifiasm ONT achieving the highest contig N50 and longest contig, and NextDenovo achieving the lowest number of contigs (Table 1). The RAFT-HERRO-hifiasm and Hifiasm ONT assemblies had the most genes successfully transferred from the domestic cat assembly (Table 2), and both, as well as the NextDenovo assembly, had nine chromosomes assembled as gapless T2T contigs (Fig. 1, Table 3). The previously published tiger haplome assembly had a single chromosome that was assembled T2T as a single contig, and the domestic cat assembly had none (Table 3). Following homology-based scaffolding, the RAFT-HERRO-hifiasm and Hifiasm ONT assemblies had 17 of the 19 tiger chromosomes as T2T scaffolds, which is a substantial improvement on the current tiger haplome assembly (Table 3).

**Table 3:** Number of scaffolds / contigs in each assembly with both, one or no telomeres

Assembly	Both telomeres	One telomere	No telomeres
Domestic cat reference	3 (0)	7 (13)	9 (388)
Tiger Haplome	13 (1)	4 (28)	2 (55)
Hifiasm	3 (0)	7 (13)	9 (3948)
Hifiasm duplex	6 (0)	11 (23)	2 (833)
Flye	17 (1)	2 (34)	0 (48)
NextDenovo	15 (9)	4 (16)	0 (14)
HERRO-RAFT-Hifiasm	17 (9)	1 (17)	1 (18)
Hifiasm ONT	17 (9)	2 (18)	0 (10)

Nb. All assemblies were filtered to remove scaffolds / contigs that were not assigned to a chromosome prior to telomere assessment. Scaffold counts are given first, with contig counts in parentheses

The NextDenovo, RAFT-HERRO-hifiasm and Hifiasm ONT assemblies also covered the largest genome fraction of the domestic cat assembly (Table 2), probably because they successfully assembled more of the complex repeat regions. These repeat regions are likely also the reason these assemblies have more misassemblies, mismatches and indels than the other assemblies when compared to the domestic cat (Table 2). When compared to the tiger haplome assembly, the HERRO-RAFT-hifiasm assembly covered the largest genome fraction, closely followed by the NextDenvovo and Hifiasm ONT assemblies (Table 2). Flye had the least misassemblies and mismatches, but this is again likely because it did not cover some of the most complex parts of the genome.

HiC scaffolding of the hifiasm ONT assembly alongside comparison to the domestic cat assembly confirmed that we successfully recovered all 19 of the cat chromosomes (Figure S3, Figure S4). HiC scaffolding also suggested that the hifiasm ONT assembly did not contain any significant large misassemblies (Figure S3). The two scaffolding methods (HiC and homology-based) produced largely syntenic assemblies, with some small differences in duplicated regions and one large translocation on chromosome E1 (Figure S5).

Assemblies generated with hifiasm were substantially more computationally efficient than any other approaches we tested, and despite the absence of error correction with Flye, this was one of the least computationally efficient methods (Table S3).

### Genome annotation

De novo annotation of the hifiasm ONT assembly predicted a total of 23,737 complete genes with at least two exons and at least 200 amino acids in the resulting protein. This is slightly more than the number of genes that were successfully lifted over from the domestic cat assembly (Table 2), and similar to the number of genes identified in the domestic cat [16]. Of the 23,737 inferred proteins, 20,511 had blast hits in the SWISS-PROT database, 14,074 were significantly assigned a KO identifier and 14,935 were assigned GO terms by interproscan. A total of 3085 of the inferred proteins remained without functional annotation (see Supplementary_data_file_1.tsv for a detailed summary of all predicted genes / proteins and functional annotations).

### Structural rearrangements

We identified various potential structural rearrangements between the domestic cat and tiger genomes and between our Sumatran tiger assembly and the published tiger haplome assembly. Our Hifiasm ONT assembly corroborates the potential existence of two large inversions between domestic cats and tigers, one at the end of chromosome D4 and one at the beginning of chromosome E2 (Fig. 2). The putative inversion on chromosome D4 contained the genes UAP1L1, MAN1B1 and DPP7 and the one on chromosome E2 contained two unnamed genes (AnAmsE2_06200 and AnAmsE2_06210 in the domestic cat genome annotation). We also identified a number of potential structural rearrangements unique to the Sumatran tiger (Fig. 2, see Figure S6 for structural rearrangements across all chromosomes). In particular, a large potential rearrangement on Chromosome E1 (Fig. 2).

## Discussion

We show that error correction of raw reads makes it possible to construct a reference-quality, near T2T, de novo genome assembly using only a single, widely available sequencing technology; ONT simplex long-read sequencing. The development of error correction methods targeted specifically at Nanopore data (which contains non-random sequencing errors that violate the assumptions of many existing error correction methods) is a significant advancement in reducing the cost, complexity and computational requirements of generating T2T assemblies [11]. This approach has allowed us to construct a more complete assembly, with fewer gaps and more chromosomes with one or both telomeres than has previously been published. This Sumatran tiger assembly provides a valuable resource for conservation efforts and will facilitate further investigation into the population dynamics and adaptations of this tiger sub-species. We hope that this could inform more targeted conservation strategies. For example, providing a basis for identifying deleterious alleles, maintaining genetic variation and informing mate selection in captive populations.

Structural rearrangement of the genome is responsible for many adaptive differences between closely related species [18–20]. We identified two large putative inversions between the domestic cat assembly and the tiger haplome assembly on chromosomes D4 and E2. Our Hifiasm ONT tiger assembly shares the tiger orientation in both cases, corroborating the existence of the putative inversions and suggesting that these could be key regions involved in differentiating tigers from domestic cats. The D4 putative inversion contained three genes MAN1B1, DPP7 and UPA1L1. MAN1B1 is involved in the degradation of glycoproteins. Mutations in MAN1B1 can cause severe disability in humans [21], and mutations in the same gene family are linked to lysosomal storage disease in domestic cats, which has similar symptoms and eventually leads to death [22]. Little is known about the function of DPP7 in cats, but in humans it plays an important role in immune function, deactivating lymphocyte cells and preventing apoptosis [23]. UAP1L1 also inhibits apoptosis in humans [24, 25]. This suggests that the putative inversion contains genes involved in critical cellular processes and so could be responsible for some of the functional differences between these species. The inversion on chromosome E2 also contained genes, but the function of these genes is currently unknown. This provides a starting point for identifying differences between cat species and provides an interesting avenue for further investigation. We also detected structural rearrangements between the published tiger haplome assembly and our Hifiasm ONT assembly, which could be indicative of genomic divergence between the two tiger individuals that were sequenced, but could also reflect assembly error and so would need further investigation to draw conclusions from.

## Conclusions

Error correction of ONT long-read data greatly improves the quality of genome assembly that is possible using this single sequencing technology alone. This should make achieving highly contiguous genome assemblies simpler and more cost and computationally efficient.

## Methods

### Sample preparation and sequencing

To generate a reference-quality genome assembly for the Sumatran tiger, we used a residual blood sample that was initially collected by a qualified veterinarian, purely for clinical purposes, and subsequently donated to the University of Nottingham for genome sequencing. The blood sample was collected from an adult female Sumatran tiger at Twycross zoo, Warwickshire, UK on 7th January 2020. The tiger was anaesthetised for a root canal filling with 270 mg ketamine and 4.5 mg medetomidine via intramuscular injection via a dart rifle and then maintained with isoflurane in oxygen via an endotracheal tube during the procedure. Bloods were collected from the left jugular vein for standard biochemistry and haematology testing. Following the procedure, the anaesthesia was reversed with atipamezole intramuscular injection. Residual blood was stored in EDTA at -80 °C before being shipped on ice to the University of Nottingham for DNA extraction and sequencing.

The tiger was captive bred in France, born on 13th January 2017 and was subsequently donated to Twycross zoo. Informed consent was provided by Twycross zoo for use of the sample in this study. Following arrival of the sample at the University of Nottingham, it was stored at -20 °C for approximately a week before DNA extraction. High molecular weight genomic DNA was extracted from the blood sample using the Monarch HMW DNA Extraction Kit for Cells and Blood (NEB; T3050L), using the Frozen Blood Extraction section of the instruction manual (NEB; www.neb.com/T3050). Samples were prepared for sequencing using Oxford Nanopore’s ligation sequencing kit v14 (ONT; SQK-LSK114) or Ultra-long DNA Sequencing kit (ONT; SQK-ULK114). Sequencing was performed using four standard (simplex) PromethION flow cells (ONT; FLO-PRO114M) and three high-duplex PromethION flow cells (ONT; FLO-PRO114HD) on the PromethION 24 platform. All DNA extraction and sequencing was performed by the University of Nottingham’s next generation sequencing facility, Deep Seq.

### Read processing and assembly

For future compatibility, raw fast5 reads were converted to pod5 format using the convert function of ONT’s pod5 software. Pod5 files have been deposited in the European Nucleotide Archive (ENA) under the project PRJEB74210, accession numbers: ERR12834989 – ERR12834997. Base calling was performed using the super accurate basecaller (SUP) model version dna_r10.4.1_e8.2_400bps_sup@v4.1.0 in Dorado v0.5.3, in basecaller mode for simplex runs and duplex mode for duplex runs. The 5mCG_5hmCG flag was used to perform simultaneous methylation calling for all reads. Duplex reads were extracted from the resulting duplex bam files by filtering for the dx:i:1 tag using samtools v1.18 [26]. Bam files were then converted to fastq format using the bam2fastq tool in samtools. All reads from the simplex runs were merged into a single fastq.gz file for testing the efficacy of genome assemblers with simplex only reads. A second merged file including the extracted duplex reads as well as simplex reads was generated for comparison.

### Genome assembly

We first generated a benchmark assembly using hifiasm v0.24.0 [27–29], without the new ONT mode, using only the simplex reads. To see how much of an improvement could be made by adding duplex reads, we repeated this assembly with both the simplex and duplex reads as input. To see if new methods could improve on these assemblies, whilst using data from just a single sequencing technology, we tested various other tools using just the simplex reads. Firstly, we generated an assembly with Flye v2.9.3 [30], which is designed for genome assembly with long, error-prone reads. Uncorrected, simplex reads were passed directly to Flye using the –nano-hq flag, the minimum overlap between reads was set to 10,000 (–min-overlap 10,000) and five rounds of polishing were performed (–iterations 5). For all other parameters, the default settings were used. Secondly, we generated an assembly with NextDenovo [9], which is designed specifically to correct, then assemble long, error prone reads. NextDenovo was run with the uncorrected simplex reads as input and default settings for all parameters. Thirdly, we tested assembly using the HERRO-RAFT-hifiasm pipeline, as described in [31]. Briefly, this involved performing error correction of the simplex reads using HERRO [8]. HERRO was run using singularity v3.8.5, using the model: model_v0.1.pt. Error corrected reads and coverage estimates were generated from the HERRO corrected reads using hifiasm with the –write-ec flag. All vs. all overlaps were then obtained by running hifiasm again with the –dbg-ovec flag, on the error corrected fasta file output from the first run. Reads were then fragmented to preserve contained reads using RAFT [31], with the error corrected fasta output from the first run of hifiasm and the all vs. all overlaps paf file as input. Final assembly was then performed using the fragmented reads output by RAFT, with hifiasm v0.24.0, without ONT mode, and with a single round of correction (-r 1, rather than the default -r 3, as recommended in [31]) and default settings for all other parameters. We did also try assembly with the (HERRO) error corrected simplex, plus duplex reads in hifiasm, but this was significantly worse than with the error corrected simplex reads alone so is not shown. Finally, we generated an assembly using hifiasm in ONT mode (using the –ont flag). This is a beta testing mode, specifically designed to correct and then assemble ONT simplex reads [32]. We used uncorrected simplex reads as input and default settings for all other parameters.

Basic assembly stats were calculated using n50 v1.5.8 [33]. Quality values (QV) and error rates were estimated using merqury v1.3 [34]. The k-mer database required for merqury was built using the duplex reads, since there is no available short-read data for this individual. The different assemblies were compared using QUAST v5.3.0 [35], with the most recent domestic cat assembly, AnAms1.0 (accession: GCA_013340865.2) [16], as the reference. We also ran QUAST a second time using the published tiger haplome assembly P.tigris_Pti1_mat1.1, accession: GCA_018350195.2) [17] as the reference. Assembly completeness was assessed using BUSCO v5.5.0 [36] with the –mode genome setting and with the carnivora_odb10 (2024–01-08) lineage dataset, which contained 14,502 single-copy genes. Telomeric repeat regions were identified using the TeloExplorer tool from the quartet toolkit v1.2.5 [37].

We tried polishing the hifiasm ONT assembly with the duplex reads using medaka v2.1.1 [38], but the polished assembly had a significantly lower QV score compared to the unpolished assembly so we do not include it here.

### Scaffolding

In order to directly compare our assemblies to published, scaffolded tiger haplome (P.tigris_Pti1_mat1.1, accession: GCA_018350195.2) and domestic cat assemblies, and to determine the number of true chromosome level T2T contigs, we performed homology-based scaffolding for each assembly using the most closely related available assembly (the tiger haplome [17]) in RagTag v2.1.0 [39].

Homology-based scaffolding can introduce reference bias and has the potential to mask or erase structural variations and individual differences. Therefore, to further assess the quality of our hifiasm ONT assembly, we scaffolded this assembly using published Hi-C data (accession: SRR8616865) [40]. Hi-C reads were mapped to our assembly using bwa-mem v0.7.19 [41] with -SP5 flags and reads with a mapping quality < 30 were removed using samtools v1.21 [26]. Scaffolding was then conducted using YaHS v1.2.2 [42]. Scaffolds were prepared for visualisation using juicer pre and visualised using juicer v1.6 [43]. Scaffolds were aligned and renamed relative to the domestic cat assembly (AnAms1.0) using RagTag v2.1.0 [39]. Because the HiC data was from a different *Panthera tigris* individual, which could also lead to potential scaffolding issues, we also investigated the synteny between the hifiasm ONT assembly scaffolded with HiC data and the same assembly scaffolded based on homology to the tiger haplome assembly. The two assemblies were aligned using minimap2 version 2.28 [44] and genomic rearrangements were detected using SyRI version 1.7.1 [45]. Plotsr version 1.1.0 [46] was then used to visualise synteny.

### Genome annotation

Utilising annotations from a well annotated assembly of a closely related species is likely to give more complete results than de novo annotation of a novel assembly without gene expression data [47]. Therefore, we used liftoff v1.6.3 [48] to transfer annotations from the most recent version of the domestic cat assembly, AnAms1.0 [16] to each of our assemblies and to the tiger haplome assembly, which currently lacks a full genome annotation [17]. We used these liftoff annotations to construct synteny plots using the GENESPACE library v1.3.1 [49] in R v4.4.1 [50], and to identify genes present in putative chromosomal inversions. Additionally, to provide a comprehensive genome annotation for future research purposes we also performed de novo annotation of the hifiasm ONT assembly. We used Augustus v3.2.3 [51], with the species model set to human (the closest available dataset to tiger), the gene model set to complete, and no in frame stop codons allowed to predict gene structures. We then predicted functional domains and GO term annotations using interproscan v 5.59–91.0 [52], KEGG pathways using KofamScan v1.3.0 and used blastp v2.16.0 [53] to search predicted proteins against the swissprot database. Finally, we combined all annotation information into a single gff file and summary table using a custom python script (available on github: https://github.com/lldean18/OrgOne_scripts/tree/main/sumatran_tiger).

### Structural rearrangement

The domestic cat, tiger haplome and hifiasm ONT tiger assembly were aligned using minimap2 version 2.28 [44] and genomic rearrangements were detected using SyRI version 1.7.1 [45]. Plotsr version 1.1.0 [46] was then used to visualise structural rearrangements and regions of conserved synteny between the assemblies.

## Supplementary Information

Supplementary Material 1.

Supplementary Material 2.

## Data Availability

Raw pod5 sequencing trace files from the simplex and duplex ONT long-read sequencing of the Sumatran tiger were deposited in the European Nucleotide Archive (ENA) under the run accession numbers ERR12834989—ERR12834997. The final homology-scaffolded hifiasm ONT assembly (SumTig1.0) and associated annotation was deposited in the ENA under the accession number GCA_980774965. All scripts used for data analysis and plotting are publicly available on github: [https://github.com/lldean18/OrgOne_scripts].

## References

[1] Rhie A, McCarthy SA, Fedrigo O, Damas J, Formenti G, Koren S, et al. Towards complete and error-free genome assemblies of all vertebrate species. Nature. 2021;592(7856):737. 33911273 10.1038/s41586-021-03451-0 PMC8081667

[2] Logsdon GA, Vollger MR, Eichler EE. Long-read human genome sequencing and its applications. Nat Rev Genet. 2020;21(10):597–614. 32504078 10.1038/s41576-020-0236-x PMC7877196

[3] Sereika M, Kirkegaard RH, Karst SM, Michaelsen TY, Sorensen EA, Wollenberg RD, et al. Oxford Nanopore R10.4 long-read sequencing enables the generation of near-finished bacterial genomes from pure cultures and metagenomes without short-read or reference polishing. Nat Methods. 2022;19(7):823. 35789207 10.1038/s41592-022-01539-7 PMC9262707

[4] Stoler N, Nekrutenko A. Sequencing error profiles of Illumina sequencing instruments. NAR Genom Bioinform. 2021;3(1):lqab019. 33817639 10.1093/nargab/lqab019 PMC8002175

[5] Hon T, Mars K, Young G, Tsai YC, Karalius JW, Landolin JM, et al. Highly accurate long-read HiFi sequencing data for five complex genomes. Sci Data. 2020;7(1):399. 33203859 10.1038/s41597-020-00743-4 PMC7673114

[6] Koren S, Bao Z, Guarracino A, Ou S, Goodwin S, Jenike KM, et al. Gapless assembly of complete human and plant chromosomes using only nanopore sequencing. Genome Res. 2024;34(11):1919–30. 39505490 10.1101/gr.279334.124 PMC11610574

[7] Zhang H, Jain C, Aluru S. A comprehensive evaluation of long read error correction methods. BMC Genomics. 2020;21(Suppl 6):889. 33349243 10.1186/s12864-020-07227-0 PMC7751105

[8] Stanojević D, Lin D, Nurk S, Sessions PFd, Šikić M: Telomere-to-Telomere Phased Genome Assembly Using HERRO-Corrected Simplex Nanopore Reads. BioRxiv 2024.

[9] Hu J, Wang Z, Sun ZY, Hu BX, Ayoola AO, Liang F, et al. NextDenovo: an efficient error correction and accurate assembly tool for noisy long reads. Genome Biol. 2024;25(1):107. 38671502 10.1186/s13059-024-03252-4 PMC11046930

[10] Hu J, Fan JP, Sun ZY, Liu SL. NextPolish: a fast and efficient genome polishing tool for long-read assembly. Bioinformatics. 2020;36(7):2253–5. 31778144 10.1093/bioinformatics/btz891

[11] Cheng H, Qu H, McKenzie S, Lawrence KR, Windsor R, Vella M, Park PJ, Li H: Efficient near-telomere-to-telomere assembly of nanopore simplex reads. Nature 2026. 10.1038/s41586-026-10105-6 PMC13070018 41639459

[12] Wibisono HT, Pusparini W. Sumatran tiger (*Panthera tigris sumatrae*): a review of conservation status. Integrative Zool. 2010;5(4):313–23. 10.1111/j.1749-4877.2010.00219.x 21392349

[13] Wilting A, Courtiol A, Christiansen P, Niedballa J, Scharf AK, Orlando L, et al. Planning tiger recovery: Understanding intraspecific variation for effective conservation. Sci Adv. 2015;1(5):e1400175. 26601191 10.1126/sciadv.1400175 PMC4640610

[14] Liu YC, Sun X, Driscoll C, Miquelle DG, Xu X, Martelli P, et al. Genome-wide evolutionary analysis of natural history and adaptation in the world’s tigers. Curr Biol. 2018;28(23):3840. 30482605 10.1016/j.cub.2018.09.019

[15] Goodrich J, Wibisono H, Miquelle D, Lynam AJ, Sanderson E, Chapman S, Gray TNE, Chanchani P, Harihar A: Panthera tigris. The IUCN Red List of Threatened Species 2022. 2022:e.T15955A214862019.

[16] Matsumoto Y, Yik-Lok Chung C, Isobe S, Sakamoto M, Lin X, Chan TF, Hirakawa H, Ishihara G, Lam HM, Nakayama S et al: Chromosome-scale assembly with improved annotation provides insights into breed-wide genomic structure and diversity in domestic cats. J Adv Res 2024. 10.1016/j.jare.2024.10.023 PMC12789764 39490737

[17] Bredemeyer K, Hillier L, Harris A, Hughes G, Foley N, Lawless C, et al. Single-haplotype comparative genomics provides insights into lineage-specific structural variation during cat evolution. Nat Genet. 2023;55(11):1953. 37919451 10.1038/s41588-023-01548-y PMC10845050

[18] Radke DW, Lee C. Adaptive potential of genomic structural variation in human and mammalian evolution. Brief Funct Genomics. 2015;14(5):358–68. 26003631 10.1093/bfgp/elv019 PMC6278953

[19] Carpinteyro-Ponce J, Machado CA. The complex landscape of structural divergence between the drosophila pseudoobscura and D. persimilis Genomes. Genome Biol Evol. 2024;16(3):047. 10.1093/gbe/evae047 PMC10980976 38482945

[20] Ferguson S, Jones A, Murra K, Schwessinger B, Borevitz JO. Interspecies genome divergence is predominantly due to frequent small scale rearrangements in *Eucalyptus*. Mol Ecol. 2023;32(6):1271–87. 35810343 10.1111/mec.16608

[21] Rafiq MA, Kuss AW, Puettmann L, Noor A, Ramiah A, Ali G, et al. Mutations in the Alpha 1,2-Mannosidase Gene, MAN1B1, Cause Autosomal-Recessive Intellectual Disability (vol 89, pg 176, 2011). Am J Hum Genet. 2011;89(2):348–348. 10.1016/j.ajhg.2011.06.006 PMC3135808 21763484

[22] Nicholas FW, Tammen I, Hub SI: Online Mendelian Inheritance in Animals (OMIA) . In. Edited by OMIA:000625–9685; 2025.

[23] Chiravuri M, Schmitz T, Yardley K, Underwood R, Dayal Y, Huber BT. A novel apoptotic pathway in quiescent lymphocytes identified by inhibition of a post-proline cleaving aminodipeptidase: A candidate target protease, quiescent cell proline dipeptidase. J Immunol. 1999;163(6):3092–9. 10477574

[24] Xiao XX, Jiang L, Hu HL, Huang YH, Yang L, Jiao Y, et al. Silencing of UAP1L1 inhibits proliferation and induces apoptosis in esophageal squamous cell carcinoma. Mol Carcinog. 2021;60(3):179–87. 33434300 10.1002/mc.23278

[25] Yang ZY, Yang ZQ, Hu ZL, Li B, Liu DY, Chen XY, et al. UAP1L1 plays an oncogene-like role in glioma through promoting proliferation and inhibiting apoptosis. Ann Transl Med. 2021;9(7):542. 33987240 10.21037/atm-20-2809 PMC8105798

[26] Danecek P, Bonfield JK, Liddle J, Marshall J, Ohan V, Pollard MO, et al. Twelve years of SAMtools and BCFtools. Gigascience. 2021;10(2):giab008. 33590861 10.1093/gigascience/giab008 PMC7931819

[27] Cheng H, Concepcion GT, Feng X, Zhang H, Li H. Haplotype-resolved de novo assembly using phased assembly graphs with hifiasm. Nat Methods. 2021;18(2):170–5. 33526886 10.1038/s41592-020-01056-5 PMC7961889

[28] Cheng HY, Jarvis ED, Fedrigo O, Koepfli KP, Urban L, Gemmell NJ, et al. Haplotype-resolved assembly of diploid genomes without parental data. Nat Biotechnol. 2022;40(9):1332. 35332338 10.1038/s41587-022-01261-x PMC9464699

[29] Cheng H, Asri M, Lucas J, Koren S, Li H. Scalable telomere-to-telomere assembly for diploid and polyploid genomes with double graph. Nat Methods. 2024;21(6):967–70. 38730258 10.1038/s41592-024-02269-8 PMC11214949

[30] Kolmogorov M, Yuan J, Lin Y, Pevzner PA. Assembly of long, error-prone reads using repeat graphs. Nat Biotechnol. 2019;37(5):540–6. 30936562 10.1038/s41587-019-0072-8

[31] Kamath SS, Bindra M, Pal D, Jain C. Telomere-to-telomere assembly by preserving contained reads. Genome Res. 2024;34(11):1908–18. 39406502 10.1101/gr.279311.124 PMC11610600

[32] Cheng H, Qu H, McKenzie S, Lawrence KR, Windsor R, Vella M, Park PJ, Li H: Efficient near telomere-to-telomere assembly of Nanopore Simplex reads. BioRxiv 2025. 10.1038/s41586-026-10105-6 PMC13070018 41639459

[33] Telatin A, Fariselli P, Birolo G. SeqFu: a suite of utilities for the robust and reproducible manipulation of sequence files. Bioengineering (Basel). 2021;8(5):59. 34066939 10.3390/bioengineering8050059 PMC8148589

[34] Rhie A, Walenz BP, Koren S, Phillippy AM. Merqury: reference-free quality, completeness, and phasing assessment for genome assemblies. Genome Biol. 2020;21(1):245. 32928274 10.1186/s13059-020-02134-9 PMC7488777

[35] Mikheenko A, Saveliev V, Hirsch P, Gurevich A. WebQUAST: online evaluation of genome assemblies. Nucleic Acids Res. 2023;51(W1):W601–6. 37194696 10.1093/nar/gkad406 PMC10320133

[36] Simao FA, Waterhouse RM, Ioannidis P, Kriventseva EV, Zdobnov EM. BUSCO: assessing genome assembly and annotation completeness with single-copy orthologs. Bioinformatics. 2015;31(19):3210–2. 26059717 10.1093/bioinformatics/btv351

[37] Lin Y, Ye C, Li X, Chen Q, Wu Y, Zhang F, et al. QuarTeT: a telomere-to-telomere toolkit for gap-free genome assembly and centromeric repeat identification. Hortic Res. 2023;10(8):uhad127. 37560017 10.1093/hr/uhad127 PMC10407605

[38] medaka: Sequence correction provided by ONT Research. [https://github.com/nanoporetech/medaka].

[39] Alonge M, Lebeigle L, Kirsche M, Jenike K, Ou S, Aganezov S, et al. Automated assembly scaffolding using RagTag elevates a new tomato system for high-throughput genome editing. Genome Biol. 2022;23(1):258. 36522651 10.1186/s13059-022-02823-7 PMC9753292

[40] Dudchenko O, Batra SS, Omer AD, Nyquist SK, Hoeger M, Durand NC, et al. De novo assembly of the Aedes aegypti genome using Hi-C yields chromosome-length scaffolds. Science. 2017;356(6333):92–5. 28336562 10.1126/science.aal3327 PMC5635820

[41] Li H: Aligning sequence reads, clone sequences and assembly contigs with BWA-MEM. 2013.

[42] Zhou CX, McCarthy SA, Durbin R. YaHS: yet another Hi-C scaffolding tool. Bioinformatics. 2023;39(1):btac808. 36525368 10.1093/bioinformatics/btac808 PMC9848053

[43] Durand NC, Shamim MS, Machol I, Rao SS, Huntley MH, Lander ES, et al. Juicer provides a one-click system for analyzing loop-resolution Hi-C experiments. Cell Syst. 2016;3(1):95–8. 27467249 10.1016/j.cels.2016.07.002 PMC5846465

[44] Li H. Minimap2: pairwise alignment for nucleotide sequences. Bioinformatics. 2018;34(18):3094–100. 29750242 10.1093/bioinformatics/bty191 PMC6137996

[45] Goel M, Sun HQ, Jiao WB, Schneeberger K. SyRI: finding genomic rearrangements and local sequence differences from whole-genome assemblies. Genome Biol. 2019;20(1):277. 31842948 10.1186/s13059-019-1911-0 PMC6913012

[46] Goel M, Schneeberger K. Plotsr: visualizing structural similarities and rearrangements between multiple genomes. Bioinformatics. 2022;38(10):2922–6. 35561173 10.1093/bioinformatics/btac196 PMC9113368

[47] Mira-Jover A, Graciá E, Giménez A, Fritz U, Rodríguez-Caro RC, Bourgeois Y. Taking advantage of reference-guided assembly in a slowly-evolving lineage: application to. Plos One. 2024;19(8):e0303408. 39121089 10.1371/journal.pone.0303408 PMC11315351

[48] Shumate A, Salzberg SL. Liftoff: accurate mapping of gene annotations. Bioinformatics. 2021;37(12):1639–43. 33320174 10.1093/bioinformatics/btaa1016 PMC8289374

[49] Lovell JT, Sreedasyam A, Schranz ME, Wilson M, Carlson JW, Harkess A, et al. GENESPACE tracks regions of interest and gene copy number variation across multiple genomes. Elife. 2022;11:e78526. 36083267 10.7554/eLife.78526 PMC9462846

[50] Team RC: R: A language and environment for statistical computing. In. Vienna, Austria: R Foundation for Statistical Computing; 2021.

[51] Stanke M, Diekhans M, Baertsch R, Haussler D. Using native and syntenically mapped cDNA alignments to improve de novo gene finding. Bioinformatics. 2008;24(5):637–44. 18218656 10.1093/bioinformatics/btn013

[52] Jones P, Binns D, Chang HY, Fraser M, Li WZ, McAnulla C, et al. InterProScan 5: genome-scale protein function classification. Bioinformatics. 2014;30(9):1236–40. 24451626 10.1093/bioinformatics/btu031 PMC3998142

[53] Sayers EW, Beck J, Bolton EE, Brister JR, Chan J, Connor R, et al. Database resources of the national center for biotechnology information in 2025. Nucleic Acids Res. 2024;53(D1):D20–9. 10.1093/nar/gkae979 PMC11701734 39526373

