# Supplementary material for: The tiger who came to T2T: Telomere-to-Telomere genome assembly of the Sumatran tiger (Panthera tigris sumatrae) using nanopore simplex reads
Source: BMC Genomics. 2026 Mar 21;27:339. doi: 10.1186/s12864-026-12703-0 (PMC13047762; doi:10.1186/s12864-026-12703-0)
Supplement: Supplementary file 2 — Supplementary Material 2. [file 12864_2026_12703_MOESM2_ESM.pdf]

SUPPLEMENTARY INFORMATION FOR:

The tiger who came to T2T: telomere-to-telomere genome assembly of the Sumatran tiger (*Panthera tigris sumatrae*) using a single sequencing technology

Laura Louise Dean[1], Nadine Holmes[1], Phillipa Dobbs[2] and Matthew Loose[1]

[1]School of Life Sciences, The University of Nottingham, University Park, Nottingham, NG7 2RD, UK

[2]Twycross Zoo, Atherstone, Warwickshire, CV9 3PX, UK

Supplementary tables

Table S1. BUSCO gene counts

Assembly	Complete (%)	Complete single copy	Complete duplicated	Fragmented (%)	Missing (%)
Domestic Cat reference	95.4	94.3	1.1	0.8	3.8
Tiger haplome	95.2	94.3	0.9	0.8	4.0
Hifiasm	56.2	55.0	1.2	1.6	42.2
Hifiasm duplex	91.1	87.1	4.0	1.0	7.9
Flye	95.3	94.4	0.9	0.7	4.0
NextDenovo	95.3	94.4	0.9	0.7	4.0
HERRO-RAFT-hifiasm	95.4	94.4	1.0	0.7	3.9
Hifiasm ONT	95.3	94.1	1.2	0.8	3.9

BUSCO gene counts were obtained using BUSCO v5.5.0 with the carnivora_odb10 lineage dataset (2024-01-08, 14,502 single-copy genes).

Table S2. Haplotype assembly statistics

Assembly	Total length (bp)	Number of contigs	N50	N90	Longest contig (bp)	Shortest contig (bp)
Hifiasm ONT Primary	2,463,154,435	95	142,172,726	44,068,790	238,981,921	271
Hifiasm ONT Haplome 1	2,379,532,969	193	82,667,907	12,803,716	221,276,263	271
Hifiasm ONT Haplome 2	2,309,294,093	177	42,589,943	7,080,255	238,981,922	31,318
RAFT-HERRO-hifiasm Primary	2,452,787,153	114	139,794,700	28,923,347	220,606,598	23,868
RAFT-HERRO-hifiasm Haplome 1	2,442,692,345	327	102,016,318	19,952,474	197,406,936	14,809
RAFT-HERRO-hifiasm Haplome 2	2,450,505,858	214	105,621,075	22,827,509	196,964,928	22,794

bp: base pairs.

Table S3. CPU hours used to generate assemblies from raw base-called reads

Assembly	CPU hrs pre-assembly error correction	CPU hrs assembly	Total CPU hrs
Hifiasm	0	338.45	338.45
Hifiasm duplex	0	289.67	289.67
Flye	0	2835.92	2835.92
NextDenovo	0	3691.87	3691.87
HERRO-RAFT-hifiasm	192.13	528.51	720.64
Hifiasm ONT	0	415.33	415.33

CPU: core processing units, hrs: hours.

Supplementary figures

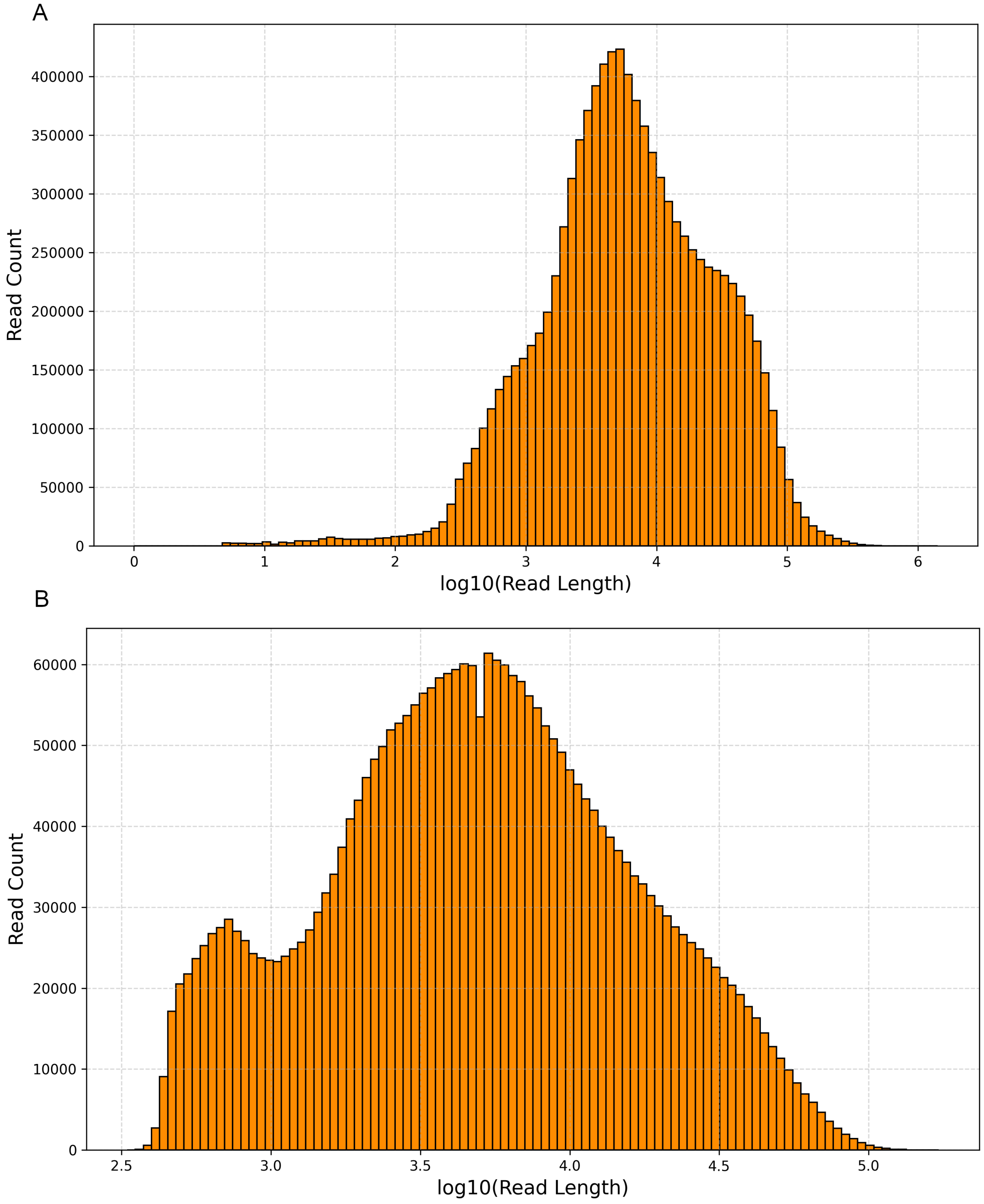

Figure S1. Read length histograms for (a) all simplex runs and (b) all duplex reads extracted from duplex runs.

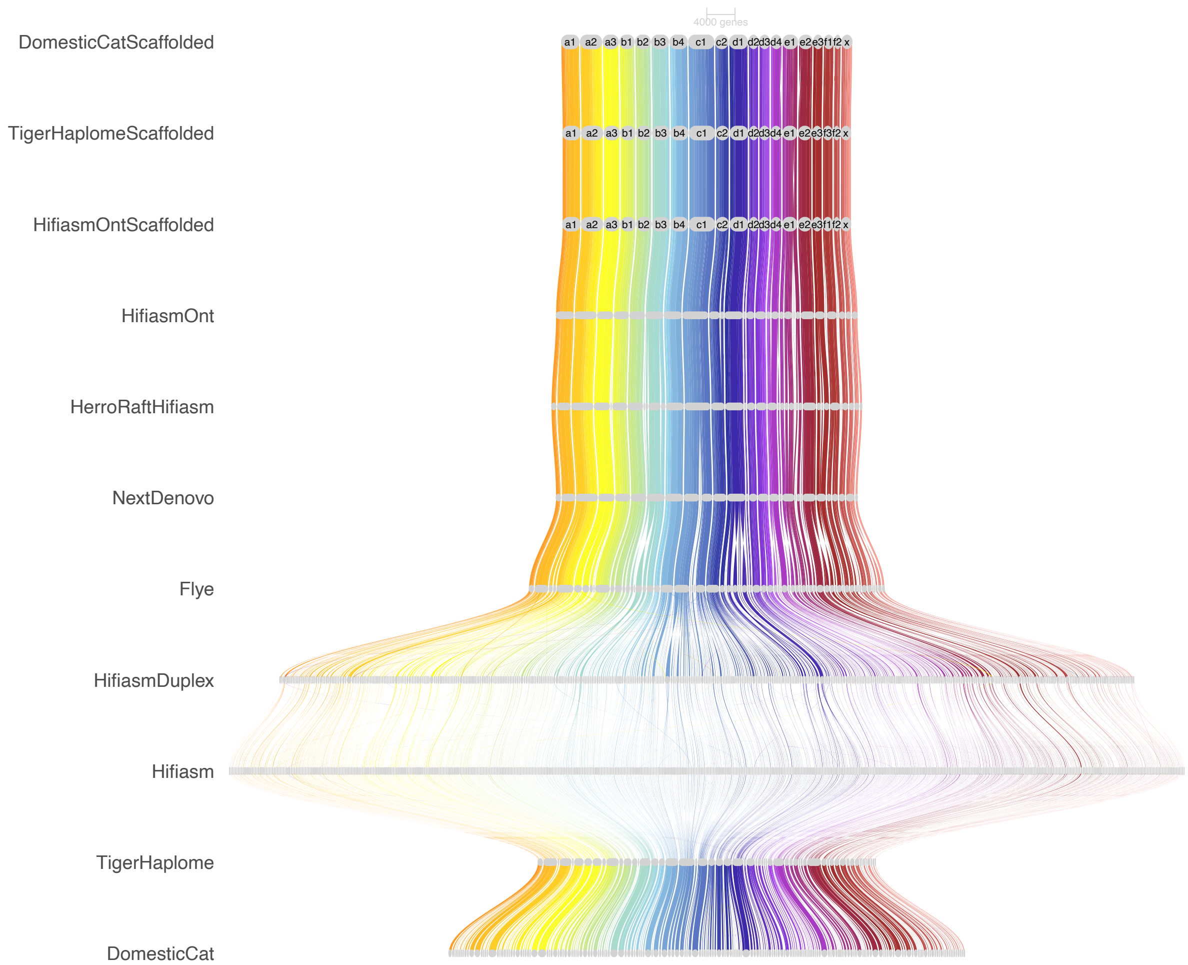

Figure S2. Synteny plot of published domestic cat and tiger haplome assemblies alongside our hifiasm ONT tiger assembly scaffolded with HiC data and all other contig-level tiger assemblies generated in this manuscript. Chromosome names are given for scaffolded assemblies.

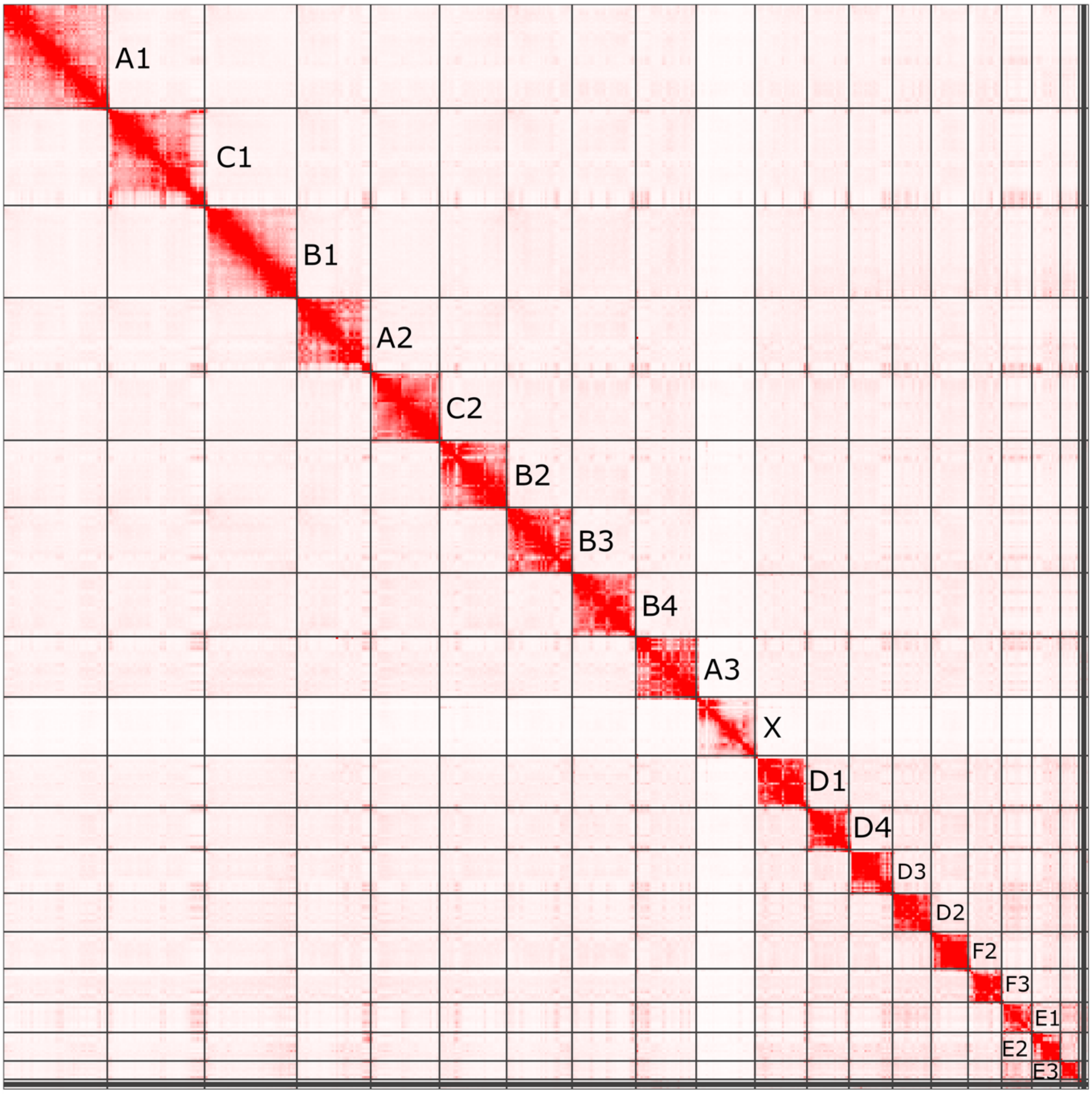

Figure S3. Hi-C contact map for the Hifiasm ONT tiger assembly. Scaffolds are labelled according to homology to the domestic cat genome.

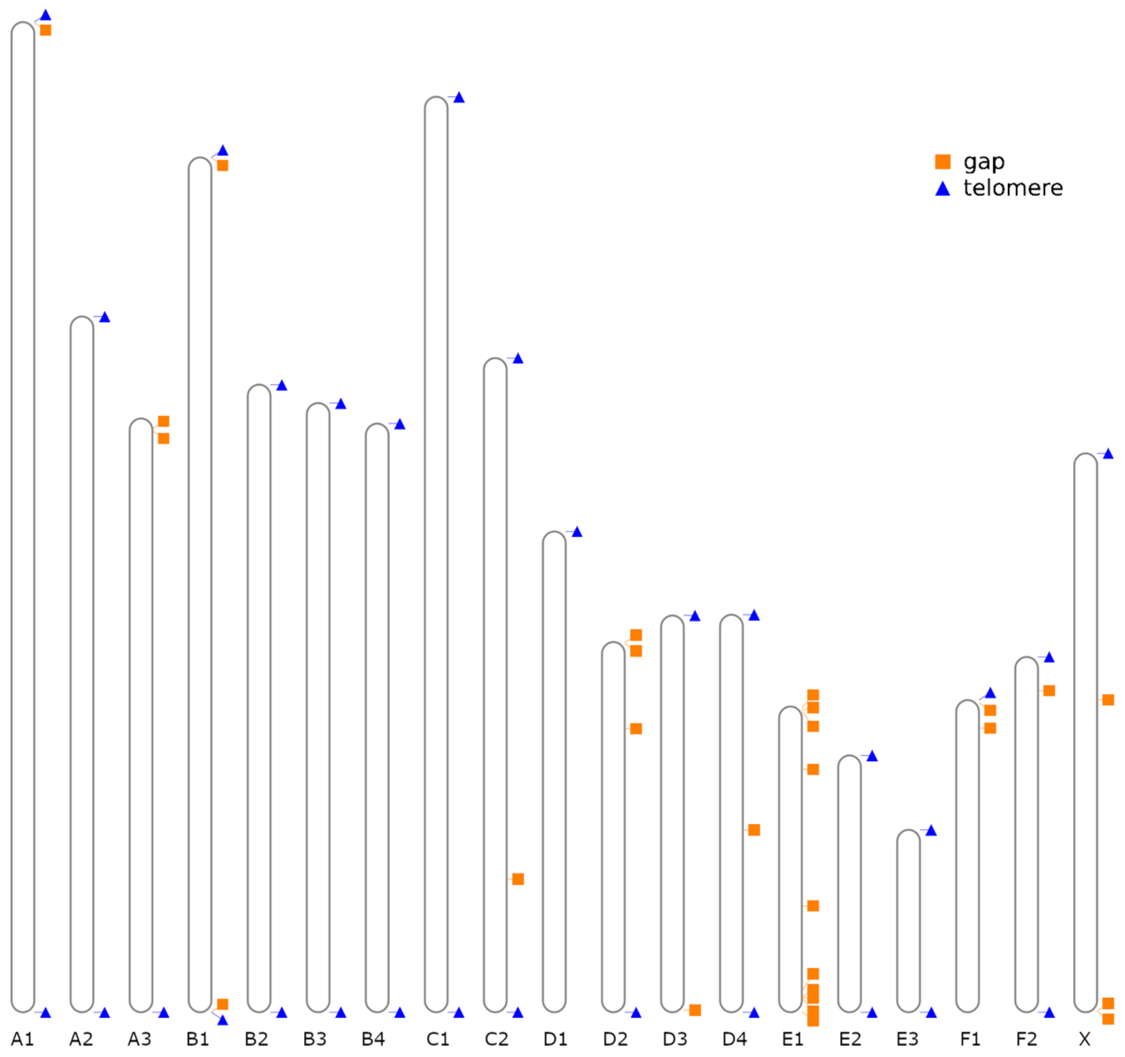

Figure S4. Locations of telomeric repeat sequences and gaps in the hifiasm ONT assembly, scaffolded with HiC data. Scaffolds are named according to homology to the domestic cat chromosomes. Unplaced contigs were removed for clarity.

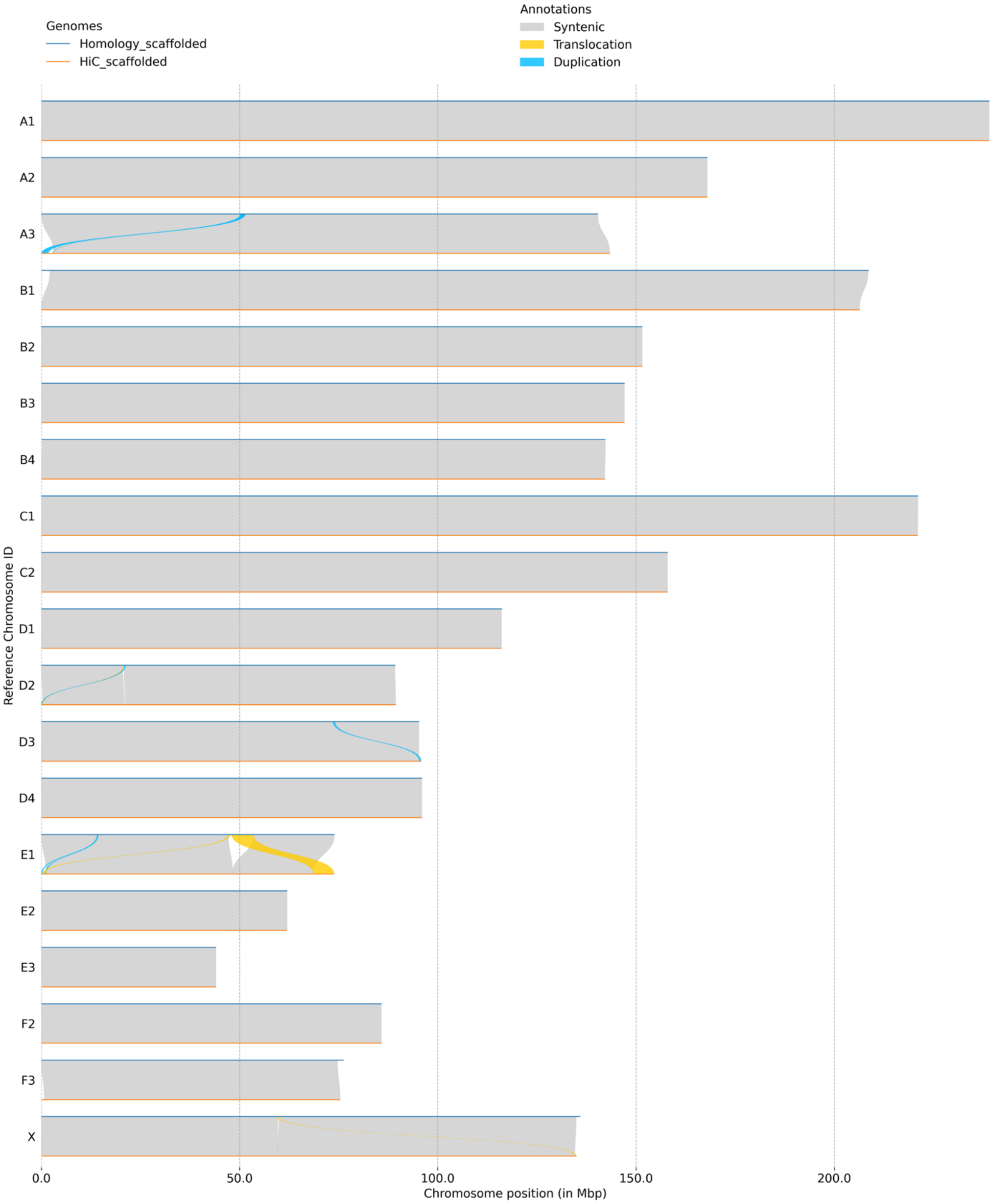

Figure S5. Synteny between the hifiasm ONT assembly scaffolded based on homology to the tiger haplome assembly and the same assembly scaffolded with HiC data from another tiger individual.

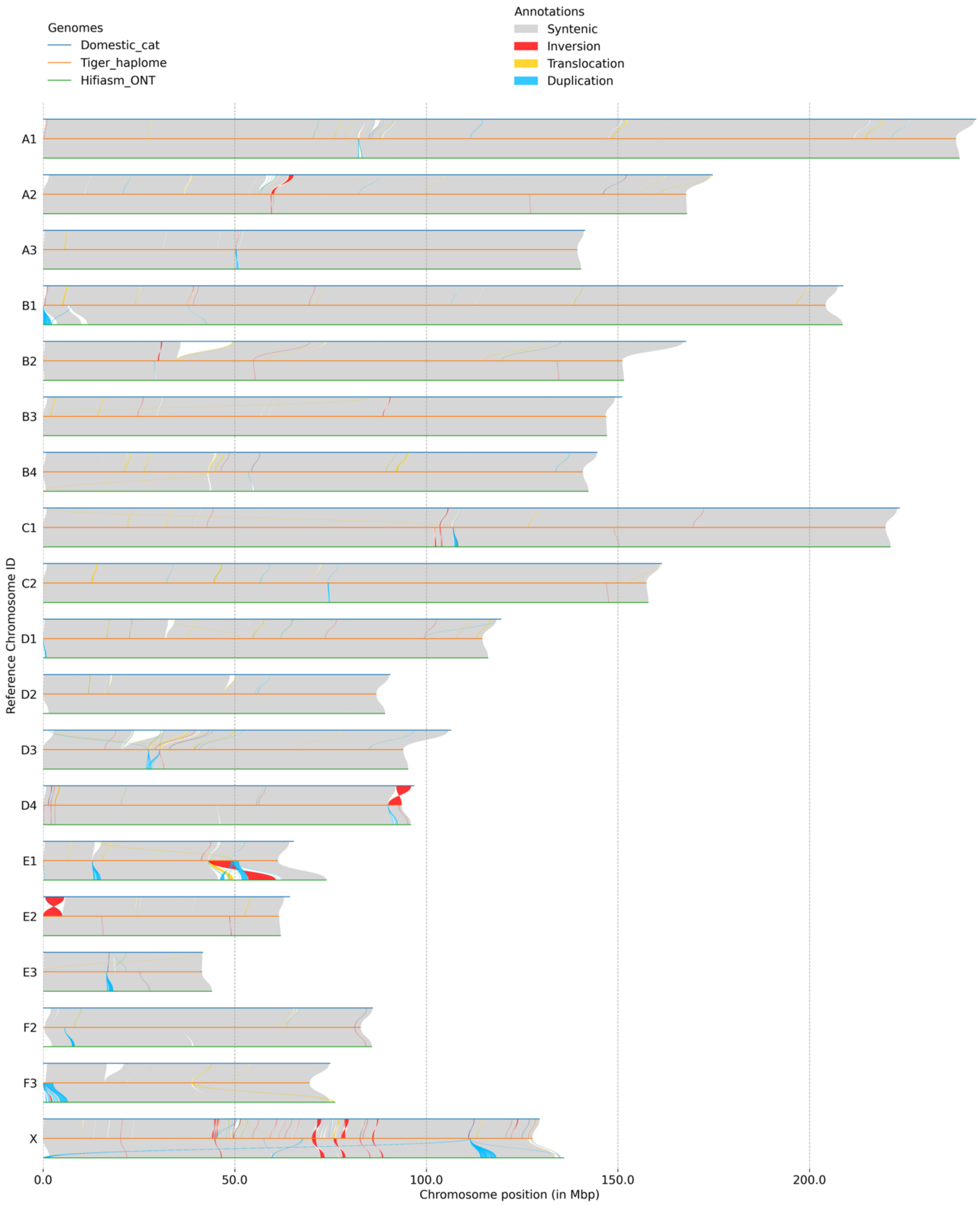

Figure S6. Chromosomal rearrangements between domestic cat, tiger haplome and hifiasm ONT assemblies across all chromosomes.
